# Individuals with bipolar disorder have a higher level of uric acid than major depressive disorder: a case–control study

**DOI:** 10.1038/s41598-021-97955-4

**Published:** 2021-09-15

**Authors:** Zhe Lu, Yingtan Wang, Guanglei Xun

**Affiliations:** 1 Cheeloo College of Medicine, Shandong University, 44# Wenhua Western Road, Jinan, 250012 China; 2 Peking University Sixth Hospital, Institute of Mental Health, Peking University, Beijing, 100191 China; 3 Department of Mental Health, Jining Medical University, 133# Hehua Road, Beihu New District, Jining, 272067 China; 4 Shandong Mental Health Center, 49# Wenhua Eastern Road, Jinan, 250014 China

**Keywords:** Psychology, Medical research

## Abstract

At present, no well-established biomarkers were ever found to distinguish unipolar depression and bipolar disorder (BD). This study aimed to provide a clearer comparison of UA levels between BD and major depressive disorder. Peripheral UA of 119 patients with BD in acute stage (AS) and 77 in remission stage (RS), and 95 patients with UD in AS and 61 in RS were measured, so were 180 healthy controls. UA levels in BD group were higher than UD and HC groups regardless of the AS or RS, while differences in UA levels between UD group and HC group were not significant. Differences in UA levels of BD-M (bipolar mania/hypomania) were higher than BD-D (bipolar depression) subgroups, and UA levels of BD-M and BD-D subgroups were higher than UD and HC groups. The comparison of number of participants with hyperuricemia among groups confirmed the above results. There were no significant differences in UA levels of between drug-use and drug-free/naïve subgroups. UA could distinguish BD and UD significantly both in acute and remission stage. The study suggests patients with BD had a higher level of UA than UD, especially in mania episode. UA may be a potential biomarker to distinguish BD from UD.

## Introduction

Bipolar disorder (BD) is a serious mental disorder with a low diagnosis rate, resulting from that the onset of BD is often characterized by a depressive episode, which is similar in presentation to unipolar depression (UD)^1^. Due to misdiagnosis, inappropriate treatment with antidepressants without concomitant mood stabilizers results in switching to mania or hypomania and repeated attacks of depression^2^. A recent study showed that family history of BD, early age at onset of the first depressive episode (< 25 years), postpartum depressive episodes, rapid onset of depressive episodes, worse response to antidepressants and the presence of psychotic symptoms or atypical depressive symptoms might be the most consistent clinical predictors of BD^3^. However, no laboratory or imaging markers are identified to allow for a diagnosis of BD or distinguishing between BD and UD.

The purinergic system is a critical neurotransmitter system with the end product of Uric acid (UA), which involves the occurrence and development of mental illness^4^. It has been proved that increased levels of UA are associated with the accelerated purinergic transformation^5^. UA acts on neurons presynaptically and postsynaptically and specific receptors in the glial cell membrane that can affect other neurotransmitters’ activities involved in the pathophysiological process of mood disorders, including dopamine, gamma-aminobutyric acid, glutamate and serotonin^6^.

In the late nineteenth century, researchers found that some patients with gout and hyperuricemia suffered from mood disorders and were relieved after receiving lithium treatment. Since then, the relation between UA and mood disorders has raised the hypothesis of purinergic system dysfunction^7^. Recent studies showed that the highest UA levels were observed in patients with BD compared with other mental disorders and healthy controls (HC)^8–11^, and elevated UA levels were associated with impulsivity, excitatory behavior, irritability, hyperthymia temperament and severe manic symptoms^6,12^. While the lowest UA levels were observed in patients with UD, suggesting that UA may be a potential biomarker for distinguishing between BD and UD. Besides, patients with BD have an increased risk of gout^13^, while allopurinol, an inhibitor of xanthine oxidase used to treat and prevent gout, can be used as an add-on therapy for patients with BD to reduce manic symptoms^14^. Some studies also implied that compared with bipolar depression and remission, the highest UA levels were observed in the manic episode, indicating that UA may be a status marker of manic episodes rather than a trait marker^15–17^. However, similar results were not detected in similar studies. Studies by Salvadore et al. and Gültekin BK et al. showed that UA levels were higher in patients with BD than in healthy controls but not associated with the severity of mania. Furthermore, some studies showed there were no statistically significant differences in UA levels between BD and UD, neither did to healthy controls^18–20^.

Previous studies on UA of patients with BD and UD are limited and conflicting. The present study aimed to conduct a clearer comparison of UA levels between BD and UD.

## Materials and methods

### Subjects and participants

The study protocol was approved by the Clinical Research Ethics Committee of Shandong Mental Health Center and is compliant with the Code of Ethics of the World Medical Association (Declaration of Helsinki). Informed written consent was obtained from all participants or their legal guardians after a complete and extensive description.

We conducted this study at the Shandong Mental Health Center form May 2018 to May 2019, inpatients and outpatients aged from 18 to 60 years with the Diagnostic and Statistical Manual of Mental Disorders, fifth edition (DSM-5) diagnosis of BD or UD were recruited. Furthermore, healthy individuals with no family history of psychiatric disorders were enrolled in the study as the control group.

Inclusion criteria for patients: (1) meet the bipolar disorder or major depressive disorder criteria based on DSM-5; (2) age 18–60 years, han Chinese; (3) understand research content and provide written informed consent.

Inclusion criteria for healthy controls (HC): (1) without any mental disorders and family history of mental disorders; (2) age 18–60 years, han Chinese; (3) HAMD-17 < 7, YMRS < 6; (4) understand research content and provide written informed consent.

The exclusion criteria for all participants were as follows: (1) Combined with organic brain diseases or brain trauma. (2) Hypertension, diabetes, gout or liver, kidney, biliary, and other physical diseases or abnormal renal and liver function. (3) Combined with other mental disorders. (4) Positive in urine pregnancy test or lactating females. (5) Modified electroconvulsive therapy treatment within 4 weeks, or long-acting antipsychotics treatment within 6 months; (6) Taking antioxidants or neurotrophic drugs within 12 weeks before and during enrollment.

All participants received an interview by a psychiatric postgraduate (Zhe Lu), the diagnosis was confirmed by at least two experienced psychiatrists based on DSM-5.

### Evaluation instruments and measurement

Demographic and clinical information of participants were collected by the self-designed case report form, which including age, sex, history of smoking, family history of psychiatric disorders, number of mood episodes, duration of disease, and whether with psychotic symptoms.

Serum UA levels and lipid indices (total cholesterol, CHOL; triglyceride, TG; high-density lipoprotein, HDL; low-density lipoprotein, LDL) test as part of routine blood checks was performed during the inpatient stays and the regular return visit of outpatients, while serum UA levels and lipid indices test of healthy individuals in this study was performed after enrollment. The assay was prepared as follows: 5 mL of fasting venous blood samples were drawn from all participants. According to the manufacturer’s instructions, serum levels of UA were detected by Roche Cobas C702 automatic biochemical analyzer (Swiss Roche Diagnostics Co., Ltd.). In Shandong Mental Health Center, the normal range of serum UA values has been standardized as 208–428 µmol/L in males and 155–357 µmol/L in females.

### Statistical analysis

All of the data were analyzed by using IBM SPSS Statistics for Windows, Version 26 (Chicago Inc., USA). All measurement data were inspected for normality by Kolmogorov–Smirnov test. Kruskal–Wallis one-way analysis of variance (ANOVA) was performed to compare the differences of age, onset age, number of mood episodes, duration of disease, LDL, HDL and TG among 3 groups. One-way ANOVA was used to compare CHOL among 3 groups. Chi-square test or Fisher’s exact test was conducted to analyze sex, history of smoking, positive family history and whether with psychotic symptom. Differences of UA were tested by analysis of covariance (ANCOVA), with age, sex, age of onset, mood episode numbers, duration of disease, whether with psychotic symptom and lipid indices as covariates to control confounding factors between BD and UD groups; age, sex, lipid indices as covariates among 3 groups. Bonferroni test as the post-hoc multiple comparison was used to identify the differences among 3 groups. Receiver operating characteristic (ROC) analysis was applied to see the possibility to use UA as a biomarker.

## Results

### Demographic and clinical data

The study included 119 BD patients in acute stage (AS) and 77 in remission stage (RS) and included 95 UD patients in AS and 61 in RS as well as 180 subjects in the HC group. Differences of sex among 3 groups were not significant whether on AS or RS. Age of BD was lower than UD (*P* < 0.001) and HC (*P* < 0.001) groups in acute stage, while the difference between UD and HC groups was not significant; on remission stage, there were no significant differences between BD and UD groups, as well as between HC and UD groups, while the age of BD group was lower than HC group (*P* = 0.001). Duration of illness and mood episode times in BD group were higher than UD group whether on AS or RS. The differences in smoking history and family history between BD group and UD group were not significant whether on AS or RS. Patients with psychotic symptoms in BD group were more than UD group. HDL of BD was lower than HC (*P* = 0.009, after Bonferroni test) groups in acute stage, while the difference between UD and HC groups, as well as between HC and UD groups were not significant; on remission stage, HDL of UD group was higher than BD and HC groups. LDL and CHOL of BD and UD groups were lower than HC groups, while the differences between UD and BD groups were not significant. There were no significant differences on TG among 3 groups on acute stage, while TG of HC was lower than BD and UD groups on remission stage (Table 1).

**Table 1:** Demographic characteristic and clinical data of participants (median (IQR25-75)/mean ± SD).

	BD		UD		HC n = 180	*F*_*1*_*/Z*_*1*_*/χ*^*2*^_*1*_	*P*_*1*_	*F*_*2*_*/Z*_*2*_*/χ*^*2*^_*2*_	*P*_*2*_
	AS n = 119	RS n = 77	AS n = 95	RS n = 61					
Sex (male/female)	61/58	37/40	43/52	28/33	90/90	0.837	0.658	0.326	0.850
Age (years)	30 (22,40)	31 (22.43)	37 (25,49)	34 (23,49)	34 (20,44.75)	22.481	< 0.001	12.638	0.002
Smokers/non-smokers	21/98	14/63	19/76	11/50	NA	0.192	0.661	0.001	0.982
Family history (positive/negative)	22/97	17/60	23/72	17/44	NA	1.042	0.307	0.615	0.433
Duration of illness (months)	60 (19,111)	50 (15,123)	20 (6,66)	15 (5,63)	NA	12.586	< 0.001	9.443	0.002
Mood episode numbers	3 (2,5)	3 (2,5)	1 (1,3)	1 (1,3)	NA	47.302	< 0.001	21.920	< 0.001
Psychotic symptom (yes/no)	40/79	NA	16/79	NA	NA	7.691	0.006	NA	NA
Medication (with/without)	62/57	NA	50/45	NA	NA	0.006	0.938	NA	NA
CHOL (mmol/L)	4.31 ± 0.87	4.37 ± 0.89	4.41 ± 0.83	4.34 ± 0.92	4.68 ± 0.85	7.886	< 0.001	5.393	0.005
TG (mmol/L)	1.14 (0.72,1.81)	1.55 (1.17,2.43)	1.21 (0.75,1.63)	1.4 (1.03,1.925)	1.02 (0.8025,1.3575)	2.247	0.325	40.611	< 0.001
HDL (mmol/L)	1.17 (1.03,1.40)	1.21 (1.06,1.48)	1.2 (1.07,1.45)	1.42 (1.285,1.79)	1.255 (1.1,1.4975)	8.862	0.012	19.494	< 0.001
LDL (mmol/L)	2.45 (1.98,2.9)	2.22 (1.195,2.66)	2.33 (2.03,3.07)	2.48 (1.985,2.89)	2.75 (2.38,3.1975)	17.975	< 0.001	33.682	< 0.001

BD, bipolar disorder; UD, unipolar depression; HC, healthy control; AS, acute stag; RS, remission stage. CHOL, total cholesterol; TG, triglyceride; HDL, high-density lipoprotein; LDL, low-density lipoprotein; SD, Standard deviation; IQR, Interquartile range.

_1_, comparison among 3 groups in acute stage.

_2_, comparison among 3 groups in remission stage.

### Differences in UA levels among BD, UD, and HC group in acute stage

There were significant differences in UA levels and number of participants with hyperuricemia among three groups. *Post-hoc* analysis showed that UA levels and number of participants with hyperuricemia in the BD group were higher than UD and HC group adjusted by bonferroni test, while differences in UA levels and number of participants with hyperuricemia between UD and HC group were not significant (Table 2).

**Table 2:** UA levels of participants in AS (mean ± SD, μmol/L).

	BD	BD-M	BD-D	UD	HC	*F*_*1,a*_	*P*_*1,a*_	*F*_*1,b*_	*P*_*1,b*_	*F*_*2*_	*P*_*2*_
	n = 119	n = 64	n = 55	n = 95	n = 180						
UA	354.02 ± 88.75	367.84 ± 92.92	337.93 ± 81.54	282.13 ± 77.98	296.27 ± 68.77	25.024	< 0.001	19.242	< 0.001	29.365	< 0.001

						*χ*^*2*^_*3*_	*P*_*3*_	*χ*^*2*^_*4*_	*P*_*4*_		
HPUA (yes/no)	30/89	20/44	10/45	6/89	11/169	28.628	< 0.001	33.463	< 0.001		

UA, uric acid; HPUA, hyperuricemia; BD-M, mania/hypomania; BD-D, bipolar depression; UD, unipolar depression; HC, healthy control; SD, Standard deviation.

_1_, age, sex, history of smoking, family history, age of onset, mood episode numbers, duration of disease, whether with psychotic symptom and lipid indices as covariates between BD and UD groups.

_2_, age, sex, history of smoking and lipid indices as covariates among 3 groups.

_3_, compare the number of participants with HPUA among BD, UD and HC groups.

_4_, compare the number of participants with HPUA among BD-M, BD-D, UD and HC groups.

_a_, comparison between BD and UD groups.

_b_, comparison among BD-M, BD-D and UD groups.

**Figure 1:**
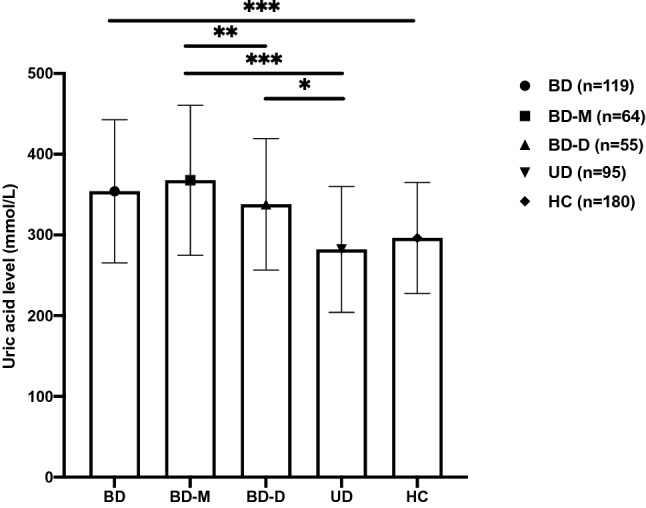
UA levels of participants in acute stage. UA of BD group was higher than UD and HC groups, while the difference between UD group and HC group was not significant. UA of BD-M subgroup was higher than BD-D subgroup and UD group, and UA of BD-D subgroup level was higher than UD group. BD, bipolar disorder; BD-M, mania/hypomania; BD-D, bipolar depression; UD, unipolar depression; HC, healthy control; UA, uric acid. *P < 0.050; **P < 0.010; ***P < 0.001.

Afterward, the BD group was divided into bipolar mania/hypomania (BD-M, n = 64) subgroup and bipolar depression (BD-D, n = 55) subgroup to be compared with the UD group. There were significant differences among the 3 groups. The *post-hoc* test showed that differences on UA levels and number of participants with hyperuricemia of BD-M subgroup were higher than BD-D (*P* = 0.002) subgroup and UD group (*P* < 0.001), and UA levels of BD-D subgroups were higher than UD group (*P* = 0.034), while the differences in number of participants with hyperuricemia between BD-D subgroup and UD group were not significant (Table 2, Fig. 1).

### Differences in UA levels among BD, UD and HC group in remission stage

Significant differences in UA levels were detected among three groups. The *post-hoc* test showed that UA levels and number of participants with hyperuricemia in BD group were higher than UD and HC group, while differences in UA levels and number of participants with hyperuricemia between UD and HC group were not significant (Table 3).

**Table 3:** UA levels of participants in RS (mean ± SD, μmol/L).

	BD (n = 77)	UD (n = 61)	HC (n = 180)	*F*_*1*_	*P*_*1*_	*F*_*2*_	*P*_*2*_
UA	364.17 ± 91.11	295.84 ± 75.96	296.27 ± 68.77	10.824	0.001	25.714	< 0.001

				*χ*^*2*^_*3*_	*P*_*3*_		
HPUA (yes/no)	24/53	4/57	11/169	33.755	< 0.001		

UA, uric acid; HPUA, hyperuricemia; UD, unipolar depression; HC, healthy control; SD, Standard deviation.

_1_, age, sex, history of smoking, family history, age of onset, mood episode numbers, duration of disease, whether with psychotic symptom and lipid indices as covariates between BD and UD groups.

_2_, age, sex, history of smoking and lipid indices as covariates among 3 groups.

_3_, compare the number of participants with HPUA among BD, UD and HC groups.

### Effects of treatment on UA levels

#### Drug-use subgroup vs. drug-naïve/free subgroup

Patients in acute stage were divided into drug-use subgroup and drug-naïve/free subgroup (mania and depression unmedicated first episode or no treatment was used within eight weeks). There were no significant differences on UA between drug-use and drug-free/naïve subgroups whether in BD group or UD group (Table 4).

**Table 4:** UA levels of drug-use and drug-naïve/free subgroups (mean ± SD, μmol/L).

	Drug-use	Drug-naïve/free	*F*	*P*
**BD (62/57)**	369.35 ± 85.82	337.33 ± 88.75	1.897	0.171
BD-M (34/30)	379.65 ± 84.67	354.47 ± 101.24	0.141	0.709
BD-D (28/27)	356.86 ± 87.07	318.30 ± 71.76	2.446	0.125
UD (50/45)	299.26 ± 78.59	263.09 ± 73.53	1.466	0.229

Age, sex, history of smoking, family history, age of onset, mood episode numbers, duration of disease, whether with psychotic symptom and lipid indices as covariates.

UA, uric acid; BD-M, mania/hypomania; BD-D, bipolar depression; UD, unipolar deprssion; SD, Standard deviation.

#### BD-M vs. BD-D vs. UD in the drug-use subgroup

In the drug-use subgroup, the differences among 3 groups were significant (*F* = 8.570, *P* < 0.001), the *post-hoc* test showed there were no significant differences in UA between BD-M and BD-D subgroups (*P* = 0.227), as well as between BD-D and UD groups(*P* = 0.080), while UA levels of BD-M group were higher than UD group (*P* < 0.001).

#### BD-M vs. BD-D vs. UD in drug-naïve/free subgroup

In drug-naïve/free subgroup, the differences among 3 groups were significant (*F* = 10.267, *P* < 0.001), there were no significant differences in UA levels between BD-D and UD groups (*P* = 0.217), but UA levels of BD-M groups were higher than UD (*P* < 0.001) and BD-D groups (*P* = 0.027).

### ROC analysis of UA as a biomarker to distinguish BD and UD

**Figure 2:**
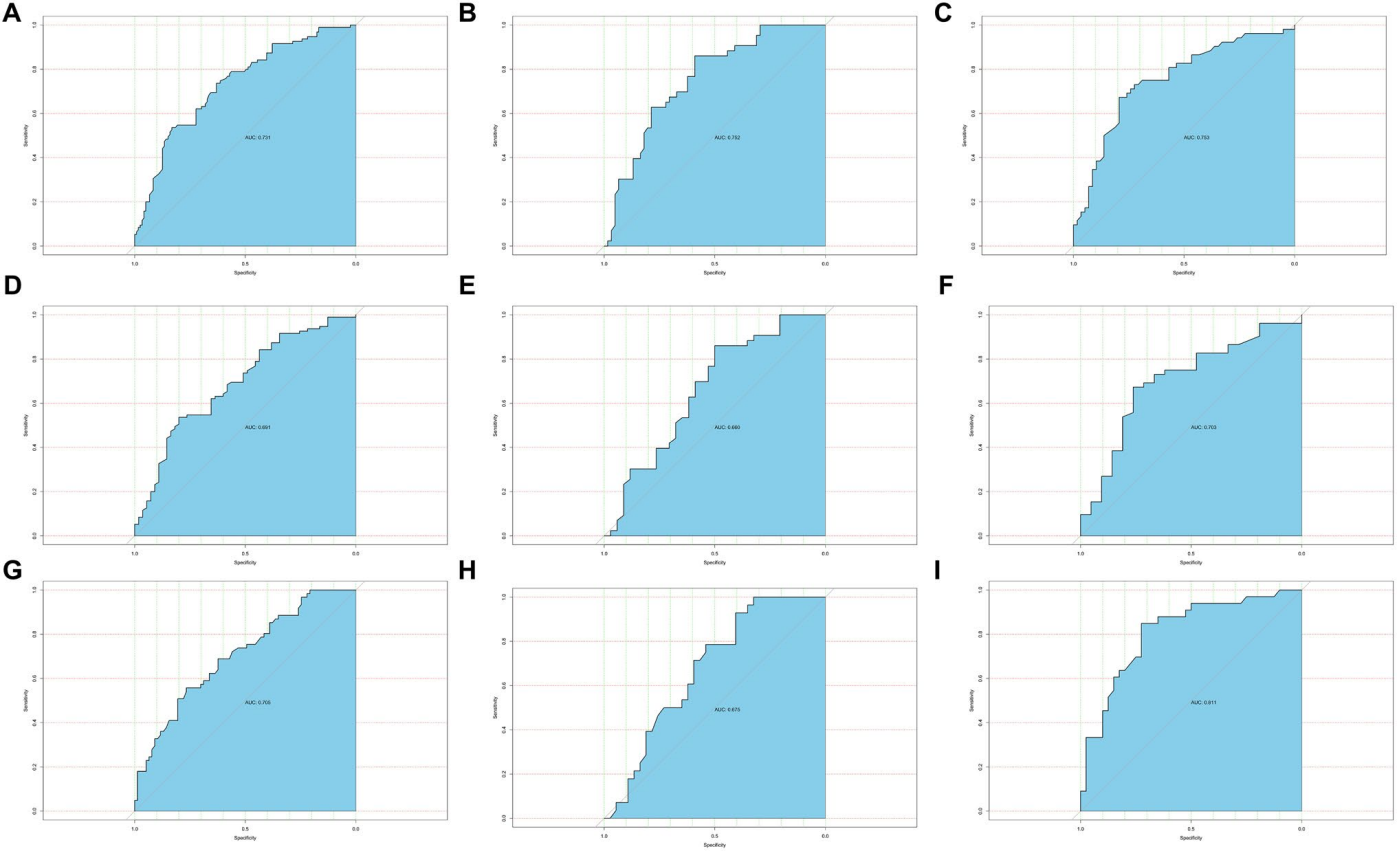
Predictive effect of the UA level. (**A**–**C**) respectively showed the results of ROC analysis, which indicated that the UA could significantly distinguish BD and UD at acute stage, in all subjects, in male subjects and in female subjects. (**D**–**F**) respectively showed the results of ROC analysis, which indicated that the UA could significantly distinguish BD-D and UD at acute stage, in all subjects, in male subjects and in female subjects. (**G**–**I**) respectively showed the results of ROC analysis, which indicated that the UA could significantly distinguish BD and UD at remission stage, in all subjects, in male subjects and in female subjects. BD, bipolar disorder; BD-D, bipolar depression; UD, unipolar depression; UA, uric acid.

UA level could significantly distinguish the BD group and UD group (area under curve: all subjects, 0.731; male subjects, 0.752; female subjects, 0.753) in acute stage, as well as BD-D group and UD group (area under curve: all subjects, 0.691; male subjects, 0.660; female subjects, 0.703). In remission stage, the UA could also significantly distinguish the BD group and UD group (area under curve: all subjects, 0.705; male subjects, 0.675; female subjects, 0.811) (Fig. 2).

## Discussion

In the study, UA levels in the BD group were higher than UD and HC groups, whether in acute or remission stage. Nevertheless, a recent study indicated that UA levels in UD were lower than HC; a possible reason was the heterogeneity of subjects in the UD group because the UA diagnosis is only based on clinical symptoms at present while some patients with BD often begin with depression. It was further confirmed by a recent study that the higher UA levels might be a predictor of BD^21^. The previous study showed that sex was an important factor that could affect UA levels^19^, but we analyzed separately by sex and got similar results.

The purinergic system is involved in neurodevelopment and pathophysiological processes of psychotic disorders, such as the process of genesis, differentiation on neurocyte and inflammation of neuro-glial cell, and so on^22–25^. Purinergic receptors can be divided into P1 and P2 receptors according to their biochemical and pharmacological properties^26^. P1 receptors can regulate plasticity of synapse and the release of neurotransmitters^24,25,27,28^, while P2 receptors are closely related to embryonic neural development^29^. The dysfunction of the purinergic system result from any causes may lead to psychotic disorders. UA, as the end product of the purinergic system, is in connection with some physiological functions, including sleep, motor, cognitive function, appetite, and social activities, as well as the pathophysiology of mood disorders^6,12^. Additionally, UA is also related to specific traits, including driving and disinhibition, which is very common in BD. It is also noticed that the peripheral UA levels are consistent with that in the central nervous system^30,31^.

Beyond that, UA is also a selective antioxidant whose level is considered as a marker of oxidative stress, and results in this study indicated that patients with BD might have a higher oxidative stress level. Moreover, in this study, we divided the acute patients with BD into BD-M and BD-D subgroups, with results showing that UA levels of both subgroups were higher than UD group, and UA levels of BD-M group were higher than BD-D group. However, there were no significant differences between BD-D and UD group on a number of patients with hyperuricemia. It suggested that patients with mania episode might have a higher level of oxidative stress.

In order to detect the effects of treatment on UA levels, we divided the acute patients into drug-use and drug-naïve/free subgroups. It was observed that the differences on UA levels between 2 subgroups were not significant, which suggested that UA might be a steady biomarker to distinguish BD and UD.

As the comparation of demographic data in Table 1, the difference of age among three groups was significant, moreover, we did a partial correlation analysis, which controls the influence of the diagnosis and sex, the result showed that the association between age and UA was significantly negative. The previous study also showed that age was negatively correlated with the UA level^32^. To eliminate the influence of confounding factors, we set the age as covariates when we conducted the comparation.

**Figure 3:**
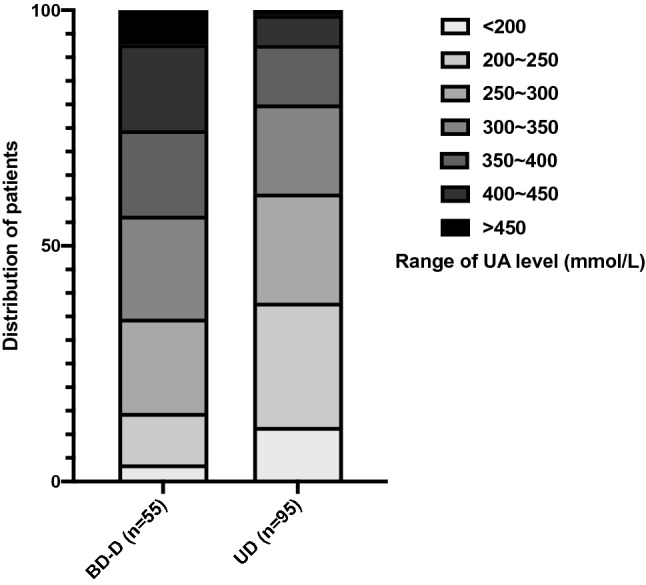
Distribution of patients in UA interval (every 50 μmol/L of UA change). UD group included the higher percent of patients with high level of UA than BD-D subgroup. BD-D, bipolar depression; UD, unipolar depression; UA, uric acid.

We draw a figure which showed the distribution of numbers of patients in the interval of UA (every 50 μmol/L of UA change), it clearly showed that the UD group included the higher percent of patients with high level of UA than BD-D subgroup (Fig. 3). To see the possibility to use UA as a biomarker clinically, we conducted a ROC analysis, the result showed that the UA could distinguish BD and UD significantly both in acute and remission stage, which indicated that the UA might be a potential biomarker to distinguish BD from UD.

There are some limitations to this study. Firstly, diet is an affecting factor to UA levels, but this study did not strictly control the diet. Secondly, mediation analysis indicated that metabolic syndrome, triglyceride, and abdominal perimeter could affect UA levels, although it could not fully explain the correlation between UA and BD^8^, we collected the lipid indices and control the confounders, but biochemical indicators like hepatorenal function and indexes of glycometabolism were not collected, which may affect the UA. Thirdly, we did not evaluate the severity of the disease because we aimed to compare the difference among different mood states, it was difficult to add the severity of disease as the covariate when conduct the comparison. A previous study showed that UA levels were positively correlated with the severity of mania^9^, but recent studies indicated that there was no significant correlation between UA and severity of mania^18,33^, which is calling for more strictly designed prospective studies to explore the relation between UA and severity of the disease. Finally, although we divided acute patients into drug-use and drug-naïve/free subgroup, the effect of different kinds of mood stabilizers on UA levels are diverse, such as lithium^34^ and carbamazepine may decrease UA levels of BD patients, while valproates seemly have the opposite effect^35^, and the effect of antidepressants, physiotherapeutic and psychotherapy on UA levels were not yet discussed.

In conclusion, this study observed that UA levels in BD were higher than UD and HC, especially in mania episode, which provide further evidence on the relation between the purinergic system and pathogenesis of BD. Moreover, UA levels may be a potential biomarker to distinguish BD from UD. In the future, a strict-design, larger-sample prospective study is required to confirm this conclusion.

## Data Availability

The datasets used and/or analyzed during the current study are available from the corresponding author on reasonable request.
